# Local and systemic innate immune response to secondary human peritonitis

**DOI:** 10.1186/cc12895

**Published:** 2013-09-12

**Authors:** Florence Riché, Etienne Gayat, Corinne Collet, Joaquim Matéo, Marie-Josèphe Laisné, Jean-Marie Launay, Patrice Valleur, Didier Payen, Bernard P Cholley

**Affiliations:** 1 Departement d'Anesthésie-Réanimation, Hôpital Lariboisière, AP-HP, 2 rue Ambroise Paré, 75010 Paris, France; 2 U942, INSERM, Hôpital Lariboisière, 2 rue Ambroise Paré, 75010 Paris, France; 3 Service de Biochimie et de Biologie Moléculaire, Hôpital Lariboisière, AP-HP, 2 rue Ambroise Paré, 75010 Paris, France; 4 Service de Chirurgie Digestive, Hôpital Lariboisière, AP-HP, 2 rue Ambroise Paré, 75010 Paris, France; 5 Université Paris Diderot, 5 rue Thomas Mann, 75013 Paris, France; 6 Service d'Anesthésie-Réanimation, Hôpital Européen Georges Pompidou, AP-HP, 20 rue Leblanc, 75015 Paris, France; 7 Université Paris Descartes - Sorbonne Paris Cité, 12 rue de l'Ecole de Médecine, 75005 Paris, France

## Abstract

**Introduction:**

Our aim was to describe inflammatory cytokines response in the peritoneum and plasma of patients with peritonitis. We also tested the hypothesis that scenarios associated with worse outcome would result in different cytokine release patterns. Therefore, we compared cytokine responses according to the occurrence of septic shock, mortality, type of peritonitis and peritoneal microbiology.

**Methods:**

Peritoneal and plasma cytokines (interleukin (IL) 1, tumor necrosis factor α (TNFα), IL-6, IL-10, and interferon γ (IFNγ)) were measured in 66 patients with secondary peritonitis.

**Results:**

The concentration ratio between peritoneal fluid and plasma cytokines varied from 5 (2 to 21) (IFNγ) to 1310 (145 to 3888) (IL-1). There was no correlation between plasma and peritoneal fluid concentration of any cytokine. In the plasma, TNFα, IL-6, IFNγ and IL-10 were higher in patients with shock *versus *no shock and in nonsurvivors *versus *survivors (*P *≤0.03). There was no differential plasma release for any cytokine between community-acquired and postoperative peritonitis. The presence of anaerobes or *Enterococcus *species in peritoneal fluid was associated with higher plasma TNFα: 50 (37 to 106) *versus *38 (29 to 66) and 45 (36 to 87) *versus *39 (27 to 67) pg/ml, respectively (*P *= 0.02). In the peritoneal compartment, occurrence of shock did not result in any difference in peritoneal cytokines. Peritoneal IL-10 was higher in patients who survived (1505 (450 to 3130) *versus *102 (9 to 710) pg/ml; *P *= 0.04). The presence of anaerobes and *Enterococcus *species was associated with higher peritoneal IFNγ: 2 (1 to 6) *versus *10 (5 to 28) and 7 (2 to 39) *versus *2 (1 to 6), *P *= 0.01 and 0.05, respectively).

**Conclusions:**

Peritonitis triggers an acute systemic and peritoneal innate immune response with a simultaneous release of pro and anti-inflammatory cytokines. Higher levels of all cytokines were observed in the plasma of patients with the most severe conditions (shock, non-survivors), but this difference was not reflected in their peritoneal fluid. There was always a large gradient in cytokine concentration between peritoneal and plasma compartments highlighting the importance of compartmentalization of innate immune response in peritonitis.

## Introduction

Secondary peritonitis is a severe compartmentalized infectious insult characterized by a rapid response of innate immunity leading to a major inflammatory process. The initial response is usually followed by a post-injury depression of innate immunity in various types of sepsis [1-4]. However, there is a paucity of data regarding systemic and local innate immune responses during peritonitis in humans and on their relation to prognosis [5-8]. The outcome of secondary peritonitis has been shown to be influenced by several clinical and bacteriological features of the disease. We recently reported that two or more micro-organisms in peritoneal fluid culture, intra-peritoneal anaerobes, yeasts, or *Enterococcus *species were associated with worse prognosis. Other groups have suggested that postoperative peritonitis was also associated with worse outcome [9-11], a finding that our own observations did not corroborate [12]. Whether or not these features are associated with differential immune responses is unknown. Therefore, the aims of this study were: 1) to characterize the inflammatory mediator response (pro-inflammatory: IL-1, TNFα, IL-6, IFNγ ; and anti-inflammatory: IL-10) in the peritoneal and blood compartments of patients with secondary peritonitis; 2) to evaluate the potential differences in mediator profiles between shocked and non-shocked patients, survivors and non survivors and according to the type of peritonitis (community-acquired or post-operative); and 3) to look at the potential impact of peritoneal microbiological findings on mediator release.

## Materials and methods

Over a period of two years, all patients with secondary peritonitis admitted in our unit (surgical intensive care unit, Lariboisière University Hospital) were prospectively screened for peritoneal and plasma cytokine measurements. The study was approved by our Institutional Review Board (N° IRB00006477, Comité d'Ethique de la Recherche Biomédicale du GHU Nord).

Plasma cytokine measurements were performed on leftovers of blood from routine daily samples and patients were informed during follow-up, but the need for written informed consent was waived. This cohort was a subgroup of a larger cohort of secondary peritonitis, which was previously reported [12]. Patients were included if they were older than 18 years and if the diagnosis of secondary generalized peritonitis (community-acquired and postoperative peritoneal infection) was confirmed surgically. They were not included if they had secondary peritonitis as a result of penetrating trauma, tertiary peritonitis defined as recurrent postoperative peritonitis, primary peritonitis (medical cause of intra-abdominal infection that did not require surgery) or if they received steroids as part of their treatment.

### Diagnosis and surgical management of generalized peritonitis

By definition, all cases required laparotomy for abdominal sepsis. After incision and visual confirmation of intra-abdominal infection involving the whole peritoneal cavity, peritoneal fluid was sampled for microbiology and cytokine measurements. Abundant peritoneal lavage was then performed using sterile saline solution. None of our patients underwent open-wound management and the abdomen was not irrigated after surgery. Ostomies were systematically preferred to primary anastomosis. We did not perform planned re-laparotomy and patients were re-operated on-demand exclusively.

### Septic shock definition

Septic shock was defined according to the criteria of the Critical Care Medicine Consensus Conference as: (1) systemic inflammatory response as defined by two or more of the following: temperature higher than 38.5°C or lower than 35°C, heart rate higher than 90/minute, respiratory rate higher than 20/minute or PaCO_2 _less than 32 mmHg or need for mechanical ventilation, white blood cell count higher than 12.0 × 10^9^/L or less than 4.0 × 10^9^/L or containing more than 10% immature forms; (2) evidence of a nidus of infection; and (3) systolic blood pressure less than 90 mmHg (for at least one hour) despite adequate fluid replacement and infusion of vasopressor associated with at least two signs of perfusion abnormality (lactic acidosis, oliguria, abrupt alteration in mental status). Particular attention was paid to microbiological findings in the peritoneal fluid that were associated with worse outcome according to our previous study (that is, higher incidence of septic shock and/or death): the presence of polymicrobial cultures (that is, ≥2 micro-organisms, bacteria or fungi), anaerobes, *Enterococcus *species, and yeasts [12]. We also evaluated the impact of 'microbial synergy' (that is, association of different bacterial strains) on cytokine plasma levels [13,14].

### Cytokines measurements

Peritoneal fluid was harvested for microbiological culture and cytokine measurement immediately after opening the peritoneal cavity. Plasma cytokines were measured daily during the first three postoperative days (D1, D2, D3). In patients presenting septic shock, the first plasma samples were always obtained within 24 hours after the onset of septic shock. To evaluate the balance between pro- and anti-inflammatory mediators, four pro-inflammatory cytokines (IL-1, TNFα, IL-6 and IFNγ) as well as one anti-inflammatory cytokine (IL-10) were measured using ELISA kits (R & D Systems Europe, Lille, France) in plasma and peritoneal fluid.

### Antimicrobial therapy

Patients received antibiotics prior to anesthesia induction according to our institutional protocols. For community-acquired peritonitis, we used amoxicillin-clavulanic acid (2 g to 200 mg) associated with gentamycin (3 mg/kg) at the time of induction of anesthesia, followed by amoxicillin-clavulanic acid (1 g to 200 mg every six hours) and gentamycin (3 mg/kg daily) for five days. For postoperative peritonitis, we used piperacillin-tazobactam (4 g) with gentamycin 3 mg/kg at induction and piperacillin-tazobactam (4 g every six hours) associated with gentamycin (3 mg/kg daily) for five days. If a patient was allergic, we used gentamycin (3 mg/kg/day) associated with ornidazole (1 g) for five days. Antibiotic therapy was then adjusted to germ sensitivity, as soon as available.

### Data collection

We collected demographic and clinical data including age, gender, Simplified Acute Physiologic Score II (SAPS II), ICU length of stay, occurrence of septic shock, ICU mortality and origin of peritonitis. Microbiological and mycological results of all cultures (peritoneal fluid collected during surgery and blood samples obtained within the first 24 hours) were recorded.

### Statistical analysis

Results are expressed as median with IQR for continuous variables and as count and percentage for categorical variables. Plasma cytokine profiles during the first three days were analyzed using Friedman repeated-measures analysis of variance on ranks. Differences in cytokine profiles according to the presence or absence of shock, ICU death or nosocomial infection were tested using two-way analysis of variance on the log-transformed values of cytokine serum concentrations. The Mann-Whitney rank sum test was used to compare values at Day 1 between subgroups (community *versus *postoperative, mono- *versus *poly-microbial peritonitis) and according to the type of bacteria in the peritoneal fluid culture (anaerobes, *Enterococcus *species, yeasts). All analyses were performed using R 2.10.1 statistical software (The R Foundation for Statistical Computing, Vienna, Austria) and StatView (SAS Institute Inc, San Francisco, CA, USA).

## Results

Sixty-six patients with secondary peritonitis were studied prospectively. Patient characteristics are presented in Table 1. Twenty three patients (34%) had septic shock and eight of them died while in ICU (ICU mortality: 34%). Laparotomy was always performed within 24 hrs of septic shock onset. The incidence of shock did not reach a difference between community-acquired and post-operative peritonitis (24% *versus *48%, respectively, *P *= 0.08). The origin of peritonitis was colon (n = 20), gastro-duodenal (n = 13), post-duodenal small bowel (n = 18), biliary tract (n = 3), appendix (n = 10) and others (n = 2). The proportion of patients with polymicrobial cultures (≥2 germs) was 51% (n = 34 patients): anaerobes, 18% (n = 12), *Enterococcus *species, 20% (n = 13), and yeasts, 14% (n = 9).

**Table 1:** Patient characteristics

Patients	Number = 66
Age (years)	59 (48 to 72)
Sex ratio F/M	36 (55%)/30 (45%)
Septic shock	23 (35%)
Average length of ICU stay (days)	20 ± 24 (1 to 109)
Community-acquired/post-operative	37 (56%)/29 (44%)
SAPS II	32.5 (18 to 48)
Overall ICU mortality	8 (12%)
Mortality among septic shock	8 (34%)
Nosocomial infections	31 (47%)

Results are expressed as mean ± SD and/or (range) or as number (%). F, female; M, male; SAPS II, Simplified Acute Physiologic Score II.

Plasma and peritoneal fluid IL-1, TNFα, IL-6, IFNγ and IL-10 at day one (day of surgical procedure) and the ratio of peritoneal to plasma concentrations are presented in Table 2. Concentrations of cytokines were always higher in peritoneum than in plasma, with great variability of the peritoneal fluid/plasma ratio among cytokines (from 5 for IFNγ to more than 1,300 for IL-1). There was no correlation between plasma and peritoneal fluid concentration of any cytokine.

**Table 2:** Plasma and peritoneal fluid cytokines and peritoneal fluid/plasma ratio at Day 1 of peritonitis

	Plasma (pg/ml)	Peritoneal fluid (pg/ml)	Peritoneal/plasma ratio
IL-1	5 (5 to 8)	7,190 (1,180 to 22,670)	1,310 (145 to 3,888)
TNFα	40 (29 to 69)	262 (90 to 882)	158 (8 to 454)
IL-6	907 (289 to 2,389)	164,352 (22,859 to 328,410)	25 (3 to 75)
IFN γ	1 (1 to 3)	3 (1 to 9)	5 (2 to 21)
IL-10	43 (21 to 136)	1,135 (260 to 2,945)	25 (4 to 75)

Values are expressed as median (25^th ^to 75^th ^percentiles).

**Figure 1:**
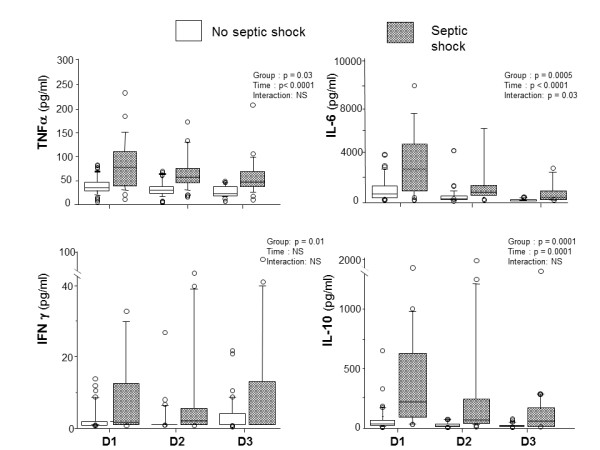
**Time course of plasma cytokines in patients with septic shock and no septic shock**.

**Figure 2:**
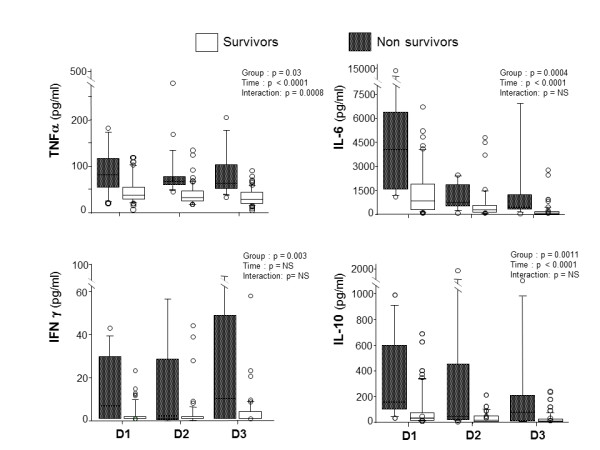
**Time course of plasma cytokines in non-survivors and survivors**.

In the plasma compartment, the IL-6 profile over the first three days differed between shock and no-shock patients (*P *< 0.03), whereas profiles of TNFα, IFNγ and IL-10 did not differ significantly (that is, no significant interaction) but inter-group and overtime differences were noted between the two groups (Figure 1). Comparing survivors *versus *non-survivors, TNFα plasma profiles had a significant interaction (*P *< 0.008) while IL-6, IFNγ and IL-10 exhibited only intergroup and overtime differences (Figure 2). Regardless of the clinical situation, plasma IL-1 did not differ among groups: shock *versus *no shock (5 (5 to 8) *versus *5 (5 to 8) pg/ml, *P *= 0.54) and survival *versus *non survival (5 (5 to 8) *versus *5 (5 to 6) pg/ml, *P *= 0.73). There was no difference in plasma cytokine concentrations between community-acquired and post-operative peritonitis. Plasma cytokine levels according to bacteriological findings (mono- *versus *poly-microbial cultures of peritoneal fluid, presence or not of anaerobes, *Enterococcus*, or yeast) are presented in Table 3. Plasma TNFα was significantly higher in the case of polymicrobial culture or when anaerobes or *Enterococcus *were present in the peritoneal fluid. Plasma IL-10 was significantly higher in the case of polymicrobial culture or when anaerobes were present in the peritoneal fluid.

**Table 3:** Plasma cytokines (pg/ml) at Day 1 according to peritoneal fluid culture

	Monomicrobial (number = 34)	Polymicrobial (number = 32)	*P *value
IL-1	5 (5 to 8)	5 (5 to 7)	0.19
TNFα	36 (26 to 52)	45 (31 to 100)	0.02
IL-6	630 (243 to 1,360)	1,500 (510 to 2,828)	0.51
IL-10	27 (5 to 67)	50 (30 to 210)	0.03
IFNγ	1 (1 to 2)	1 (1 to 3)	0.49

	**No anaerobes (number = 44) **	**Anaerobes (number = 12) **	

IL-1	5 (5 to 8)	5 (5 to 8)	0.93
TNFα	38 (29 to 66)	50 (37 to 106)	0.017
IL-6	876 (300 to 1,846)	2,755 (456 to 4,550)	0.21
IL-10	40 (17 to 90)	197 (27 to 715)	0.0005
			
IFNγ	1 (1 to 2)	1 (1 to 13)	0.12

	**No *Enterococcus sp*. (number = 43) **	***Enterococcus sp*. (number = 13) **	

IL-1	5 (5 to 7)	5 (5 to 9)	0.79
TNFα	39 (27 to 67)	45 (36 to 87)	0.02
IL-6	880 (283 to 2,618)	1,500 (630 to 2,325)	0.71
IL-10	40 (20 to 114)	50 (30 to 154)	0.17
IFNγ	1 (1 to 3)	1 (1 to 5)	0.32

	**No yeast (number = 57) **	**Yeast (number = 9) **	

IL-1	5 (5 to 8)	5 (5 to 5)	0.39
TNFα	39 (29 to 63)	52 (40 to 88)	0.54
IL-6	855 (283 to 2,282)	1,360 (1,085 to 2,580)	0.65
IL-10	40 (20 to 129)	50 (39 to 119)	0.96
IFNγ	1 (1 to 2)	2 (1 to 3)	0.62

Results are presented as median (25^th ^to 75^th ^percentiles). See text for details.

We did not find any difference in peritoneal cytokines with regard to the occurrence of shock. Only peritoneal levels of IL-10 were higher in survivors *versus *non-survivors (1,505 pg/ml (450 to 3,130) *versus *102 pg/ml (9 to 710), respectively, *P *= 0.04). Table 4 shows peritoneal cytokine levels in relation to bacteriological findings. Anaerobes and *Enterococcus *species were associated with higher IFNγ in peritoneal fluid. The presence of yeast did not result in higher plasma or peritoneal cytokine levels, but only a few patients from our cohort (n = 9) had yeast in their peritoneal fluid.

**Table 4:** Peritoneal fluid cytokines (pg/ml) at Day 1, according to peritoneal fluid culture

	Monomicrobial (number = 34)	Polymicrobial (number = 32)	*P *value
IL-1	8,194 (3,115 to 31,440)	4,400 (509 to 16,730)	0.09
TNFα	290 (89 to 1,185)	260 (93 to 695)	0.42
IL-6	72,390 (13,760 to 201,600)	236,800 (86,670 to 383,700)	0.02
IL-10	550 (243 to 4,246)	1,505 (320 to 2,610)	0.78
IFNγ	3 (1 to 23)	2 (1 to 7)	0.23

	**No anaerobes (number = 44) **	**Anaerobes (n = 12) **	

IL-1	4,682 (1,000 to 19,220)	26,000 (7,282 to 54,890)	0.04
TNFα	252 (95 to 851)	805 (88 to 1,009)	0.54
IL-6	164,400 (25,380 to 328,400)	156,800 (1,536 to 270,300)	0.44
IL-10	1,252 (386 to 2,785)	370 (167 to 4,380)	0.96
IFNγ	2 (1 to 6)	10 (5 to 28)	0.01

	**No *Enterococcus sp*. (number = 43) **	***Enterococcus sp*. (number = 13) **	

IL-1	4,850 (1,000 to 22,580)	8,528 (1,850 to 38,880)	0.33
TNFα	265 (96 to 848)	185 (87 to 1,592)	0.89
IL-6	195,600 (25,460 to 342,800)	51,400 (12,800 to 190,000)	0.12
IL-10	1,755 (403 to 3,679)	435 (167 to 480)	0.01
IFNγ	2 (1 to 6)	7 (2 to 39)	0.05

	**No yeast (number = 57)**	**Yeast (number = 9)**	

IL-1	7,859 (1,417 to 27,090)	3,010 (840 to 4,693)	0.25
TNFα	285 (90 to 953)	120 (96 to 530)	0.37
IL-6	143,300 (20,630 to 348,300)	196,800 (98,600 to 215,100)	0.88
IL-10	1,252 (358 to 2,902)	465 (40 to 2,292)	0.34
IFNγ	3 (2 to 9)	2 (1 to 4)	0.16

Results are presented as median (25^th ^to 75^th ^percentiles). See text for details

## Discussion

In this cohort of 66 patients with secondary generalized peritonitis, we observed a large gradient between peritoneal fluid and plasma concentrations of cytokines, with no correlation between peritoneal and plasma levels. Plasma TNFα, IL-6, IFNγ and IL-10 were higher in patients with shock and in non survivors and decreased over the first three postoperative days in a parallel manner. Plasma cytokines did not differ between community-acquired and postoperative peritonitis. The presence of peritoneal anaerobes or *Enterococcus *species was associated with a higher plasma level of TNFα. IL-10 plasma levels were also higher when anaerobes or polymicrobial positive culture were found. In the peritoneal compartment, there was no difference in cytokine concentration between shock and no shock patients, but there was a greater IL-10 concentration in survivors. The presence of anaerobes and *Enterococcus *species was associated with higher peritoneal IFNγ.

There are very limited data on innate immune response assessed by plasma and peritoneal inflammatory mediators in patients with secondary peritonitis. To our knowledge, only one study addressed this issue and reported a large concentration gradient for TNFα and IL-6 between peritoneal fluid and plasma in a small group (n = 17) of patients with peritonitis [6]. Our results confirm that IL-1, TNFα, IL-6, IL-10 and IFNγ are present at high concentrations in the peritoneal fluid of patients with peritonitis. This observation fits well with the concept of the 'tip of the iceberg' which suggests that plasma levels increase only after saturation of tissues within the abdominal compartment [15,16]. However, a direct intravascular stimulation of cytokine release by monocytes cannot be ruled out in some patients, especially when bacteremia occurred, since this condition is associated with higher plasma cytokines [17]. In this cohort, the proportion of positive blood cultures within the first 48 hours of peritonitis was 11/66 (16%).

We also observed that plasma levels of pro- and anti-inflammatory mediators were higher in patients with shock and in non-survivors, a finding that has been reported by several authors [1,6-8,18,19]. The fact that pro- and anti-inflammatory mediators are released simultaneously during the early phase of peritoneal sepsis confirms what was recently noted in animal models of sepsis [20] and septic patients [4,21,22]. The only data available regarding IFNγ and outcome during abdominal infection come from experimental studies and the results are conflicting. According to some authors, low plasma IFNγ is associated with increased mortality [23,24] while others reported that prophylactic inhibition of IFNγ improves survival [25,26]. Our values for plasma and peritoneal IFNγ measurements represent the first set of data in humans with GP. The plasma concentration of IFNγ was very low compared to other cytokines, in accordance with what has been observed in experimental models of peritonitis [4].

Similarly, no human data were available on IL-10 during peritonitis. Experimental studies suggest that intra-peritoneal or subcutaneous IL-10 injections reduce mortality [27], which fits well with our observation that the intra-peritoneal IL-10 concentration was greater in survivors. Since IL-10 is considered an anti-inflammatory mediator, our findings corroborate the fact that both pro- and anti-inflammatory mediators are produced simultaneously in the peritoneum of patients with peritonitis [28].

In a previous study, we found that polymicrobial cultures or anaerobes in the peritoneal fluid are associated with more frequent septic shock [12]. The role of the infecting pathogen on the innate immune response of patients with peritonitis has been poorly investigated. Controversy exists regarding a specific role of some pathogens on the pattern of the immune response. Some authors support the concept of a 'generic septic response' in which an identical immune response is triggered by any type of bacteria [29,30]. This theory has been contradicted by others who suggested that different types of germs may elicit various immune responses, despite a common pathway of activation. For example, distinct host responses to S*treptococcus pneumoniae *or *Pseudomonas aeruginosa *have been reported in experimental models of pneumonia [31]. In humans with sepsis of various origins, Gogos *et al*. recently observed that HLA-DR expression and the apoptosis rate of CD-4 and CD-8 within 24 hours of sepsis onset varied according to the underlying pathogen [32]. In rats with peritonitis, Montravers *et al*. showed that adjunction of *Enterococcus faecalis *is associated with increased mortality as well as higher levels of TNFα and IL-6 in peritoneal fluid [33,34]. In the present study, we observed that patients in whom anaerobes or *Enterococcus *species were isolated from peritoneal fluid cultures released more TNFα in their plasma than those who were infected with other strains. One of the limitations of our study is that the number of patients with specific characteristics or bacteriological findings in each subgroup is rather small, preventing any definitive conclusion to be drawn from these results. In addition, small differences must be considered cautiously as multiple comparisons were performed to compare various subgroups. However, some of the observations we made are consistent with previous experimental findings and, therefore, provide the first human data to support laboratory hypotheses.

## Conclusions

Secondary peritonitis is responsible for an acute local and systemic innate immune response involving a simultaneous release of both pro- and anti-inflammatory mediators. The most severely affected patients had higher plasma levels of all cytokines but this difference was not observed with peritoneal fluid cytokines. A large gradient in cytokine concentration was noted between the peritoneal cavity and plasma, highlighting the importance of compartmentalization of innate immune response during peritonitis.

## Key messages

• Compartmentalization of innate immune response during peritonitis is reflected by a large gradient in cytokine concentration between peritoneum and plasma.

• Higher plasma levels of pro and anti-inflammatory cytokines are associated with shock and mortality.

• Innate immune response was not different between patients with post-operative and community-acquired peritonitis.

• The infecting pathogen might influence host immune response.

## Abbreviations

CD: cluster of differentiation; ELISA: enzyme-linked immunosorbent assay; HLA-DR: human leucocyte antigen-DR; IL: interleukin; INFγ: interferon gamma; SAPS: Simplified Acute Physiologic Score; TNFα: tumor necrosis factor alpha.

## Competing interests

The authors declare that they have no competing interests, except DP who acknowledges receiving lecturing fees from Eli-Lilly, Torey Medical, Vitech Italy and Meditor in the past five years.

## Authors' contributions

FR conceived the study and its design, and drafted the manuscript. BC analyzed data and drafted the manuscript. EG performed the statistical analyses. CC and JML carried out the cytokine dosage. PV participated in study design and coordination. MJL participated in enrollment and correct routing of blood samples. JM and DP helped to interpret data and participated in manuscript drafting. All authors read and approved the final manuscript.
